# LncRNA HOTAIR-mediated MTHFR methylation inhibits 5-fluorouracil sensitivity in esophageal cancer cells

**DOI:** 10.1186/s13046-020-01610-1

**Published:** 2020-07-11

**Authors:** Shuyao Zhang, Fuchun Zheng, Liqun Zhang, Zuojun Huang, Xiaoshan Huang, Zhen Pan, Shuang Chen, Chenchen Xu, Yi Jiang, Shuyi Gu, Chengkuan Zhao, Qiuzhen Zhang, Ganggang Shi

**Affiliations:** 1 Department of Pharmacology, Shantou University Medical College, Shantou, 515041 P.R. China; 2 Department of Pharmacology, Guangzhou Red Cross Hospital, Jinan University, Guangzhou, 510220 P.R. China; 3 Pharmaceutical Laboratory, The First Affiliated Hospital, Shantou University Medical College, Shantou, 515041 P.R. China; 4 Information Section, Cancer Hospital of Shantou University Medical College, Shantou, 515031 P.R. China; 5 Department of Digestive Oncology, Cancer Hospital of Shantou University Medical College, Shantou, 515031 P.R. China

**Keywords:** Esophageal cancer, lncRNA HOTAIR, MTHFR, 5-fluorouracil, Chemosensitivity

## Abstract

**Background:**

Esophageal cancer (EC) represents one of the most aggressive digestive neoplasms globally, with marked geographical variations in morbidity and mortality. Chemoprevention is a promising approach for cancer therapy, while acquired chemoresistance is a major obstacle impeding the success of 5-fluorouracil (5-FU)-based chemotherapy in EC, with the mechanisms underlying resistance not well-understood. In the present study, we focus on exploring the role of long non-coding RNA (lncRNA) HOTAIR in EC progression and sensitivity of EC cells to 5-FU.

**Methods:**

Paired cancerous and pre-cancerous tissues surgically resected from EC patients were collected in this study. Promoter methylation of the MTHFR was assessed by methylation-specific PCR. RIP and ChIP assays were adopted to examine the interaction of DNA methyltransferases (DNMTs) with lncRNA HOTAIR and MTHFR, respectively. EC cells resistant to 5-FU were induced by step-wise continuous increasing concentrations of 5-FU. The sensitivity of EC cells to 5-FU in vivo was evaluated in nude mice treated with xenografts of EC cells followed by injection with 5-FU (i.p.).

**Results:**

We found reciprocal expression patterns of lncRNA HOTAIR and MTHFR in EC tissues and human EC cells. Interference with lncRNA HOTAIR enhanced 5-FU-induced apoptosis, exhibited anti-proliferative activity, and reduced promoter methylation of the MTHFR in EC cells. Besides, overexpression of MTHFR attenuated the acquired chemoresistance induced by overexpression of lncRNA HOTAIR in EC cells. At last, enhanced chemosensitivity was observed in vivo once nude mice xenografted with lncRNA HOTAIR-depleted EC cells.

**Conclusion:**

Together, our study proposes that pharmacologic targeting of lncRNA HOTAIR sensitizes EC cells to 5-FU-based chemotherapy by attenuating the promoter hypermethylation of the MTHFR in EC.

## Background

Esophageal cancer (EC) ranks as the eighth most prevalent cancer globally [1]. Multiple risk factors are reported to contribute its occurrence and development, including cigarette smoking, alcohol consumption, obesity, and low fruit/vegetable intake [2]. Currently, standard treatment modalities for patients with EC include surgery, radiation therapy, and chemotherapy [3]. Despite substantial advances in diagnosis and treatment, EC has overall poor prognosis, with overall 5-year survival rates ranging between 15 and 25% [4]. 5-fluorouracil (5-FU) is widely considered to be the most effective chemotherapeutic agent for treatment for EC [5]. However, esophageal tumors frequently develop resistance to 5-FU, especially in recurrence cases [6]. Therefore, there is a need to identify novel molecular targets that can facilitate the promotion of chemosensitivity of EC cells to 5-FU.

LncRNAs are a group of non-protein-coding transcripts with nucleotides lengths of 200 or more and are reported as dysregulated in tumor initiation and progression, including EC [7]. LncRNA HOX transcript antisense RNA (HOTAIR), an inhibitor of the HOXD genes, is secreted from the HOXC locus [8]. LncRNA HOTAIR, a typical lncRNA, has been implicated in the development of esophageal squamous cell carcinoma (ESCC) [9]. In particular, HOTAIR is understood to be capable of regulating cell invasiveness, migration, and apoptosis in ESCC, and thus has been proposed as a novel biomarker relevant to its diagnosis and prognosis [10]. Notably, Yan et al. have suggested that upregulation of HOTAIR is associated with chemoresistance [11]. Previous studies have revealed that alterations in DNA methylation are innate to various human cancers, including EC [12, 13]. HOTAIR is known to regulate gene expression through epigenetic modifications, including DNA methylation [14].

In addition, methylenetetrahydrofolate reductase (MTHFR), involved in 5-methyltetrahydrofolate synthesis and homocysteine remethylation, may affect the occurrence and development of cancer by directly regulating DNA methylation [15]. The role of MTHFR in EC has also been demonstrated [16]. Notably, MTHFR has been shown to affect 5-FU based chemotherapy in colorectal cancer [17]. However, the role of lncRNA HOTAIR-mediated methylation of the MTHFR promoter in the chemosensitivity of EC cells to 5-FU remains largely enigmatic. Thus, the aim of the present study was to investigate the possible effects of lncRNA HOTAIR on the chemosensitivity of EC cells to 5-FU via a potential regulation of MTHFR methylation.

## Methods

### Ethics statement

The study protocol was approved by the Ethics Committee of the Cancer Hospital of Shantou University Medical College. Written informed consent was obtained from all patients prior to enrollment. All animal experiments were conducted in accordance with the Guide for the Care and Use of Laboratory Animal by International Committees. Every effort was made to minimize the numbers and suffering of the included animals.

### Study subjects

A total of 70 EC tissue samples and adjacent normal tissues were collected from patients (43 patients < 50 years old and 27 patients ≥50 years old) diagnosed with non-specific invasive EC at the Cancer Hospital of Shantou University Medical College from April 2017 to March 2019. All included EC tissues were diagnosed pathologically as ESCC. Morphological observations and diagnosis of all cases were made by more than two deputy pathologists according to World Health Organization (WHO) classification criteria [18]. There were 47 cases with EC at grade I + II and 23 cases with EC at grade III. Tumor staging was conducted according to American Joint Committee on Cancer (AJCC) 8th Edition TNM Staging Form [19]. The patients were classified into the TNM stage of I + II (58) and the TNM stage of IIIa (12). All patients showed no lymph node metastasis (LNM), and received neither radiotherapy nor chemotherapy prior to surgery.

### Preparation of 5-FU resistant EC cell lines

Cell lines in this study were tested for mycoplasma contamination prior to experiments. The EC cell lines KYSE150, EC109, and TE-1 and human normal esophageal epithelial cell line HEEC were purchased from American Type Culture Collection (ATCC) (VA, USA). All cell lines were added with RPMI 1640 medium (Santa Cruz Biotechnology, Inc., Santa Cruz, CA, USA) supplemented with 10% FBS and 100 U/mL penicillin -streptomycin and cultured in a 5% CO_2_ incubator at 37 °C. Next, the concentration gradient method was used to construct 5-FU resistant EC cell lines (TE-1/5-FU) for three times, where 5-FU concentrations used were from 1 to 20 μg/mL, respectively. After transduction for 190 days, the concentration 5 μg/mL of 5-FU that could stabilize the drug resistance of TE-1 cells was selected for subsequent analysis.

### Cell grouping and treatment

TE-1/5-FU cells (4 × 10^5^ cells/well) were inoculated in 6-well plates. Expression vectors containing the lncRNA HOTAIR or MTHFR, two shRNAs against lncRNA HOTAIR, and their respective negative controls (empty vector, scramble shRNA) were obtained from Shanghai Sangon Biotechnology Co. Ltd. (Shanghai, China) and delivered into TE-1/5-FU cells using the lipofectamin 2000 kit according to manufactures’ instructions. 5-Aza-CdR was used to inhibit DNA methylation in cells.

### RNA isolation and quantification

Total RNA was extracted from tissues and cells using RNeasy Mini Kit (Qiagen Company, Hilden, Germany). The total RNA was reverse transcribed into cDNA using a PrimeScript RT kit (TaKaRa Biotechnology Co. Ltd., Dalian, China) according to the manufacturer’s protocol. Primer sequences of lncRNA HOTAIR, MTHFR-U, and MTHFR-M (Table 1) were designed and then synthesized by TaKaRa Biotechnology Co. Ltd. (Dalian, China). The ABI7500 quantitative PCR instrument (7500, ABI Company, Oyster Bay, NY, USA) was employed to conduct reverse transcription quantitative polymerase chain reaction (RT-qPCR). The 2^-ΔΔCt^ method was used to calculate the relative mRNA expression levels of the target genes.

**Table 1:** Primer sequences for RT-qPCR

Genes		Sequences (5′-3′)
lncRNA HOTAIR	F	GGAAAGATCCAAATGGGACCA
	R	CTAGGAATCAGCACGAAGCAAA
MTHFR-U	F	GGCTGACCTGAAGCACTTGAA
	R	AGAAAAGCTGCGTGATGATGAA
MTHFR-M	F	TGAAGGAGAAGGTGTCTGCGGGA
	R	AGGACGGTGCGGTGAGAGTG
GAPDH	F	AGAAGGCTGGGGCTCATTTG
	R	AGGGGCCATCCACAGTCTTC

*F* forward, *R* reverse

### Western blot analysis

TE-1 cells or tissue samples were lysed with radio immunoprecipitation assay (RIPA) peptide lysis buffer (BB-3209, Shanghai BestBio Co., Ltd., Shanghai, China) to extract the total protein. The proteins were separated with sodium dodecyl sulfate-polyacrylamide gel electrophoresis (SDS-PAGE) for 1 h and then transferred onto a polyvinylidene fluoride (PVDF) membrane. The membrane was incubated with rabbit anti-human primary antibody MTHFR (1: 1000, ab203785, Abcam Inc., Cambridge, MA, USA) at 4 °C overnight with GAPDH (1: 500, ab8245, Abcam Inc., Cambridge, MA, USA) used as the internal reference gene. Next, the membrane was incubated with horseradish peroxidase (HRP)-labeled goat anti-rabbit immunoglobulin G (IgG) (1: 20000; ab205718, Abcam Inc., Cambridge, MA, USA). Protein blots were visualized by ECL-associated fluorography (Merck Millipore, Billerica, MA, USA).

### Dual luciferase reporter gene assay

The MTHFR dual luciferase reporter gene vector and mutants with lncRNA HOTAIR binding site mutation (MTHFR-WT and MTHFR-MUT) were each constructed. These two reporter plasmids were co-transfected into cells that overexpressed lncRNA HOTAIR and NC plasmids. The Dual-Luciferase Reporter Assay System from Genecopoeia (D0010, Beijing Solarbio Science & Technology Co. Ltd., Beijing, China) was employed to detect the luciferase activity of MTHFR promoter region induced by lncRNA HOTAIR in EC cells. The fluorescence intensity was measured using the GLomax20/20 Luminometer (Promega Corporation, Madison, WI, USA).

### RNA-fluorescence in situ hybridization (FISH) assay

The website http://lncatlas.crg.eu/ was employed to predict the localization of lncRNA HOTAIR in TE-1 EC cells, which was identified using a FISH kit (Roche Diagnostics GmbH, Mannheim, Germany). The cells were incubated with a digoxin-labeled lncRNA HOTAIR probe (Sigma, St. Louis (MO, USA), followed by staining with 4′, 6-diamidino-2-phenylindole (DAPI) (Sigma, St. Louis, MO, USA). Then, the cells were washed with cold PBS and photographed using a confocal laser scanning microscope (FV1000, Olympus, Tokyo, Japan).

### RNA-binding protein immunoprecipitation (RIP) assay

Cell lysates were incubated with protein-G agarose beads pre-coated with anti-DNMT1 (ab13537, Abcam, Cambridge, UK), anti-DNMT3a (, ab2850, Abcam, Cambridge, UK), anti-DNMT3b (ab2851, Abcam, Cambridge, UK) or normal rabbit IgG. The resultant complexes were then incubated with 150 μL proteinase K buffer to extract protein. Total RNA was extracted using the TRIZOL method and used for RT-qPCR.

### Methylation-specific PCR (MSP) assay

Frozen EC tissues and adjacent normal tissues were obtained. DNA was extracted using the ammonia-chloroform extraction method and modified with sodium bisulfite. The modified DNA was purified using a DNA Purification Kit (Promega, Madison, WI, USA), and amplified with bisulfite-modified DNA as a template. Primers for MTHFR MSP-M and MTHFR MSP-U were synthesized by Shanghai Sangon Biotechnology Co. Ltd. (Shanghai, China). The PCR reaction conditions were 35 cycles of pre-denaturation at 95 °C for 10 min, denaturation at 94 °C for 1 min, annealing at 60 °C for 50 s, and extension at 72 °C for 10 min. The MSP results were determined as described in a previous study [20].

### Chromatin immunoprecipitation (ChIP) assay

A ChIP kit (Merck Millipore, Billerica, MA, USA) was used to detect the enrichment of DNMT1, DNMT3a, and DNMT3b within the MTHFR promoter region. TE-1 cells were treated with formaldehyde for 10 min to generate DNA-protein cross-links. Cell lysates were sonicated to generate chromatin fragments of 200–300 bp and immunoprecipitated with DNMT1 (ab13537, Abcam, Cambridge, UK), DNMT3a (ab2850, Abcam, Cambridge, UK), DNMT3b (ab2851, Abcam, Cambridge, UK), IgG as a negative control, or RNA polymerase ii antibody as a positive control. The Protein Agarose/Sepharose was used to precipitate the endogenous DNA-protein complexes, followed by de-crosslinking at 65 °C overnight. MTHFR promoter-specific primer sequences (Table 1) were used for detecting the binding of DNMT1, DNMT3a, and DNMT3b with the MTHFR promoter region.

### 5-ethynyl-2′-deoxyuridine (EdU) labeling assay

EC cells were seeded into 96-well plates (1.6 × 10^5^ cells/well), and experimental procedures were conducted according to the instructions of EdU kit (C10310, Guangzhou RiboBio Co., Ltd., Guangdong, China). Briefly, each well was added with 50 μM EdU (100 μL) at 37 °C for 4 h, followed by fixation in 4% formaldehyde at room temperature for 15 min. Next, the cells were treated with 0.2% Triton X-100 at room temperature for 5 min and 100 μL Apollo® mixture (C10338–2, Guangzhou RiboBio Co., Ltd., Guangdong, China) for 30 min, and then cultured with 100 μL Hoechst33342 (Guangzhou RiboBio Co., Ltd., Guangdong, China). DAPI was added for 30 min in order to label the cell nuclei. Cells were observed and imaged using a fluorescence microscope (Olympus, Tokyo, Japan). The Image-Pro Plus (IPP) 6.0 software (Media Cybernetics, Bethesda, MD, USA) was utilized to count the EdU-positive cells (red).

### Cell counting kit-8 (CCK-8) assay

When cell confluence reached about 80%, the cells were washed with PBS twice, and trypsinized (at 0.25%) to prepare a single cell suspension. After counting, these cells were inoculated into 96-well plates (3 × 10^3^ ~ 6 × 10^3^ cells/well) at 200 μL of suspension per well. Six wells were used as replicates of each condition. After incubation for 24 h, 48 h, and 72 h, samples were removed and incubated with 10 μL CCK-8 (VP757, DOJINDO, Kumamoto, Kyushu, Japan) for 2 h. The optical density (OD) value was measured at a wavelength of 450 nm using a microplate reader (BIOBASE-EL10A, Jinan Boxin Biotechnology Co., Ltd., Jinan, China). The cell viability curve was plotted with time-duration of incubation on the X-axis and OD value on the Y-axis. All experiments were repeated three times and the IC_50_ value was determined, which referred to the drug concentration required for cell survival. The IC_50_ value was calculated by the least square method using FORECAST function in EXCEL software.

### Flow cytometry

Propidium (PI) staining was adopted to assess the cell cycle of EC cells. Briefly, 48 h after transfection, cells were washed 3 times with cold PBS, centrifuged, and the supernatant was discarded. The cell concentration was adjusted to approximately 1 × 10^5^ cells/mL after cells were resuspended in PBS, and added with 1 mL of pre-cooled 75% ethanol (− 20 °C) to fix cells at 4 °C for 1 h, followed by centrifugation. The ice ethanol and the supernatant were discarded, and cells were added with 100 μL of RNase A in the dark, water bathed for 30 min 37 °C, and then added with 400 μL of PI (Sigma). The cells were then incubated in dark conditions at 4 °C for 30 min, and the cell cycle was determined using flow cytometry at 488 nm.

The apoptosis of EC cells was detected using an Annexin V-fluorescein isothiocyanate (FITC)/PI double staining kit (556,547, SHANGHAI SOLJA TECHNOLOGY CO., LTD., Shanghai, China). In brief, the cell suspension was incubated with 5 μL Annexin V-FITC for 15 min, followed by incubation with 5 μL PI for 5 min. FITC was detected at wavelengths of 480 nm and 530 nm and PI was detected at a wavelength of more than 575 nm using a flow cytometer (Cube6, Partec, Inc., IL, USA).

### Tumor xenografts in nude mice

A total of 48 nude mice of specific pathogen free (SPF) grade (aging 4 weeks and weighing 14–16 g) were obtained from the Medical Discovery Leader Co., Ltd. (Beijing China). A total of 2 × 10^6^ cells were mixed with 50 μL Matrigel Matrix (1: 1) and inoculated subcutaneously into the armpit of the nude mice. After 28 days, anesthesia was induced, and the nude mice were euthanized using 3% Sodium pentobarbital (1 ml/100 g, P3761, Sigma-Aldrich Chemical Company, St Louis, MO, USA), and the tumor tissues were obtained for further analysis.

### Immunohistochemistry

The expression of MTHFR was detected using peroxidase-labeled streptavidin peroxidase (SP). The paraffin samples of mouse tissues were sectioned in a continuous manner into 5 μm sections. After routine dehydration, immunohistochemistry was carried out according to routine protocol. Briefly, after treatment with 3% hydrogen peroxide at room temperature for 10 min to block endogenous peroxidase, normal non-immune animal serum was added for 10 min. The sections were treated with primary antibody rabbit anti human MTHFR (ab203785, 1: 1000, Abcam, Cambridge, UK), followed by overnight incubation at 4 °C, added with 1: 500 diluted secondary antibody (goat anti rabbit IgG) labeled with biotin, incubated at 37 °C for 20 min, and added with 50 μl streptavidin-peroxidase solution, followed by incubation at room temperature for 10 min. The sections were visualized with diaminobesidine for 5–10 min and counter-stained with hematoxylin, followed by dehydration, permeabilization, and mounting. The sections were observed under a microscope, and PBS served as NC.

### Statistical analysis

All data was analyzed using SPSS 22.0 software (IBM Corp., Armonk, NY, USA). The measurement data was expressed as mean ± standard deviation of three independent tests. The independent sample *t*-test was used to compare measurement data between two groups including gene expression between EC tissues and adjacent normal tissues. Comparison among multiple groups was conducted using one-way analysis of variance (ANOVA), followed by Tukey’s post hoc test. Repeated measures ANOVA was used to compare tumor volume among groups, followed by Tukey’s post hoc test. *p* value < 0.05 was indicative of statistical significance.

## Results

### Web-available microarray analysis indicated involvement of HOTAIR and MTHFR in EC

**Fig. 1:**
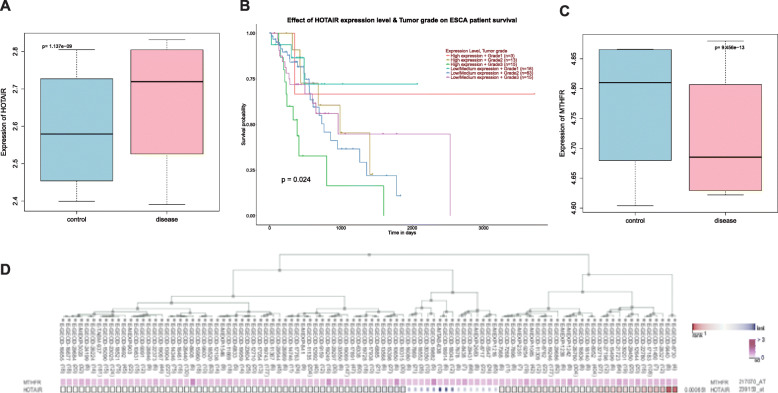
Bioinformatics analysis. **a** Box diagram of HOTAIR expression extracted from EC-related gene expression dataset GSE100942 in GEO database (https://www.ncbi.nlm.nih.gov/gds) using R language. The left blue box refers to the HOTAIR expression in normal samples and the right red box refers to the HOTAIR expression in EC samples (*p* = 1.137e-09). **b**. Survival curve of EC patients in relation to the expression of HOTAIR in the Ualcan database (http://ualcan.path.uab.edu/index.html) (*p* = 0.024). **c** Box diagram of MTHFR expression extracted from the EC-related gene expression dataset GSE100942 using R language. The left blue box refers to the HOTAIR expression in normal samples and the right red box refers to the HOTAIR expression in EC samples (*p* = 9.456e-13). **d** Co-expression analysis of lncRNA HOTAIR and MTHFR analyzed by MEM (https://biit.cs.ut.ee/mem/index.cgi) (*p* = 6.51e-04)

The EC-related gene expression dataset GSE100942 was used to predict HOTAIR expression in EC. Results showed that HOTAIR was highly expressed in EC (Fig. 1a), which was consistent with previous reports. EC-related data in the TCGA database was analyzed using ‘Ualcan’ database, and the resultant survival curve showed that HOTAIR expression was significantly correlated with survival in EC (*p* < 0.05) (Fig. 1b). MTHFR expression data were obtained from another EC-related gene expression dataset GSE100942, which showed that MTHFR was poorly expressed in EC (Fig. 1c). The co-expression analysis of lncRNA HOTAIR and MTHFR gene by MEM showed highly significant co-expression (*p* < 0.001) (Fig. 1d).

### LncRNA HOTAIR was upregulated in EC tissues and cells

**Fig. 2:**
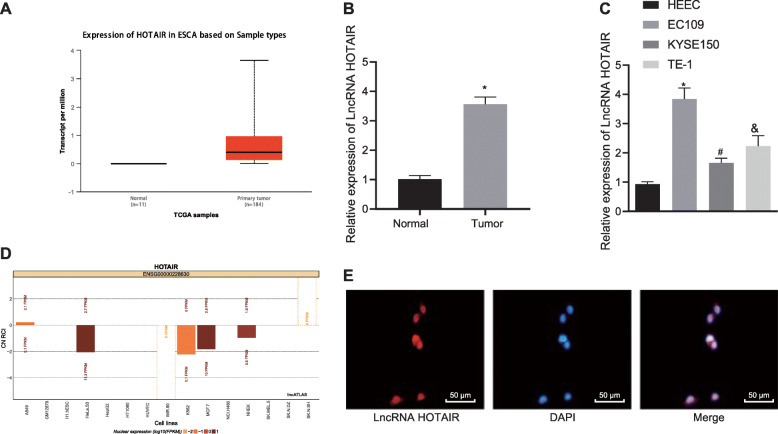
HOTAIR is upregulated in EC tissues and cell lines. **a** HOTAIR expression analyzed using the TCGA database. **b** HOTAIR expression in EC tissues (*n* = 70) and adjacent normal tissues (n = 70) determined by RT-qPCR, normalized to GAPDH. (C) HOTAIR expression in EC cell lines and HEEC determined by RT-qPCR, normalized to GAPDH. **d** The localization of lncRNA HOTAIR in TE-1 EC cells predicted using the subcellular location website (http://lncatlas.crg.eu/). **e** The localization of lncRNA HOTAIR in TE-1 EC cells verified using FISH assay (× 200). Values obtained from three independent experiments in triplicate are expressed as mean ± SD and analyzed using paired t test between EC tissues and adjacent normal tissues, and using unpaired t test between EC cells and HEEC cells. In panel **b**, * represents comparison with adjacent normal tissues, * *p* < 0.05. In panel **c**, * represents comparison with HEEC cells, * *p* < 0.05

The TCGA database revealed that HOTAIR was highly expressed in EC (Fig. 2a). The EC tissues and adjacent normal tissues from 70 patients were used to determine HOTAIR expression using RT-qPCR, which was correspondingly found to be significantly higher in EC tissues than in adjacent normal tissues (*p* < 0.05) (Fig. 2b). Furthermore, RT-qPCR displayed increased HOTAIR expression in EC cell lines EC109, KYSE150, and TE-1 in comparison to HEEC cells (*p* < 0.05) (Fig. 2c). The website ‘Lncatlas’ predicted that lncRNA HOTAIR was located in the nucleus (Fig. 2d), which was further verified using FISH (Fig. 2e). These results indicated that HOTAIR expression was elevated in EC tissues and cells and it was mainly located in the nucleus.

### Silencing of HOTAIR promoted chemosensitivity to 5-FU

**Fig. 3:**
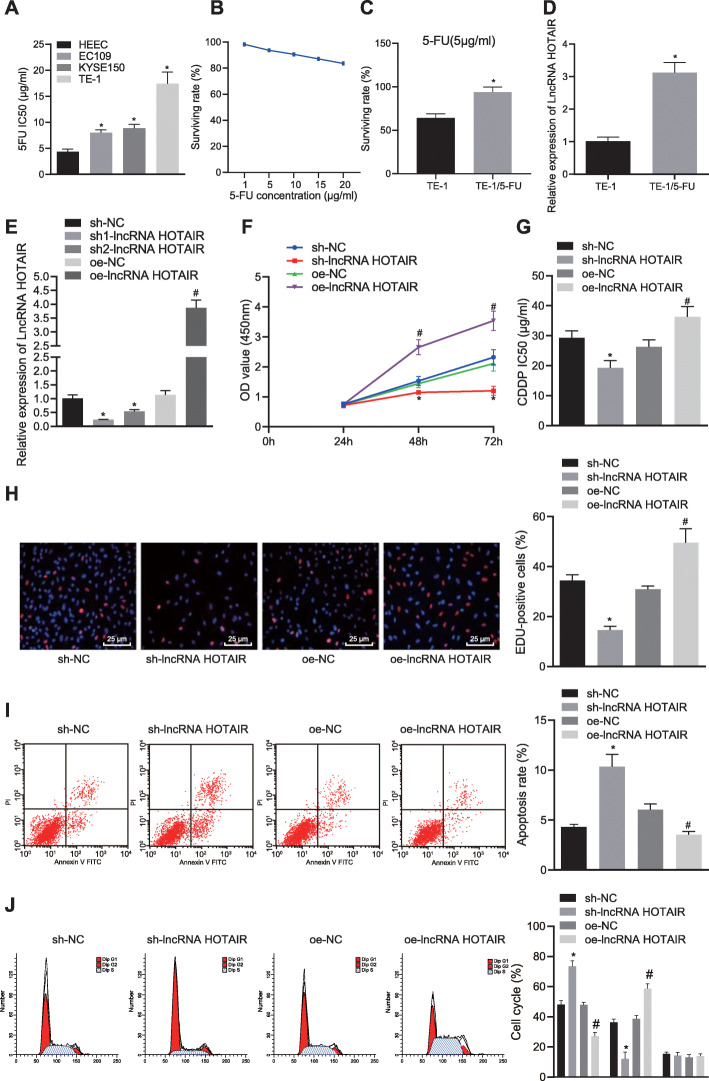
Silencing of HOTAIR facilitates chemosensitivity to 5-FU and apoptosis and reduces the proliferation of EC cells. Two shRNAs were delivered into TE-1/5-FU cells to specifically blunt HOTAIR (sh1-lncRNA HOTAIR and sh2-lncRNA HOTAIR group). Expression vectors containing the lncRNA HOTAIR were introduced into TE-1/5-FU cells (oe-lncRNA HOTAIR group). Scramble shRNA-treated cells and empty vectors-treated cells served as negative controls (sh-NC group and oe-NC group). (**a**) CCK-8 assay was performed to test the sensitivity of EC cell lines to 5-FU. (**b**) CCK-8 assay was performed to assess the surviving rate of TE-1/5-FU resistant strain in response to different concentrations of 5-FU. (**c**) CCK-8 assay was performed to assess the surviving rate of TE-1 cell line and TE-1/5-FU cell line at a 5-FU concentration of 5 μg/mL. (**d**) RT-qPCR was performed to determine the lncRNA HOTAIR expression in TE-1 cells and TE-1/5-FU cells, normalized to GAPDH. (**e**) Silencing and overexpression efficiency in TE-1/5-FU cells detected using RT-qPCR, normalized to GAPDH. (**f**) CCK-8 assay was performed to detect TE-1/5-FU cell viability following HOTAIR alternations. (**g**) CCK-8 assay was performed to measure IC_50_ value in TE-1/5-FU cells following HOTAIR alternations. (**h**) EdU assay was performed to measure cell proliferation of TE-1/5-FU cells following HOTAIR alternations (× 400) (**i**) Flow cytometry was performed to assess apoptosis of TE-1/5-FU cells following HOTAIR alternations. (**j**) Flow cytometry was performed to assess cell cycle of TE-1/5-FU cells following HOTAIR alternations. Values obtained from three independent experiments in triplicate are expressed as mean ± SD and analyzed using ANOVA followed by Tukey’s post hoc test among three or more groups. *p < 0.05 compared with TE-1/5-FU cells treated with either sh-NC or oe-NC group

In order to study the effect of lncRNA HOTAIR on drug-resistant cell lines, we used CCK-8 assay to test the sensitivity of cell lines to 5-FU, which showed that compared with KYSE150 and EC109 cell lines, the TE-1 cell line was the most resistant to 5-FU (*p* < 0.05) (Fig. 3a). Thus, TE-1 cells were selected to construct TE-1/5-FU resistant strain. 5-FU at higher concentration (from 1 μg/mL to 20 μg/mL) gradually decreased the cell surviving rate (Fig. 3b). We further used CCK-8 assay to compare the surviving rate of TE-1 cell line and TE-1/5-FU cell line at a 5-FU concentration of 5 μg/mL, and found that the surviving rate of TE-1/5-FU cell line was significantly increased relative to that of TE-1 cell line (*p* < 0.05) (Fig. 3c). To identify the relationship of lncRNA HOTAIR to drug resistance in EC, we determined HOTAIR expression in the TE-1 cell line and in the TE-1/5-FU cell line. HOTAIR exhibited significantly higher expression in the TE-1/5-FU cell line (*p* < 0.05) (Fig. 3d). Next, TE-1/5-FU cells were treated with oe-NC, sh-NC, oe-lncRNA HOTAIR, sh1-lncRNA HOTAIR, and sh2-lncRNA HOTAIR plasmids to determine the effects of knockdown or overexpression of lncRNA HOTAIR on chemosensitivity. The findings revealed that as compared with the TE-1/5-FU cells treated with sh-NC plasmids (Fig. 3e), HOTAIR expression was decreased in TE-1/5-FU cells treated with sh1-lncRNA HOTAIR and sh2-lncRNA HOTAIR plasmids, with a more pronounced decline upon treatment with sh1-lncRNA HOTAIR (*p* < 0.05). Thus, TE-1/5-FU cells treated with sh1-lncRNA HOTAIR were selected for further experiments. HOTAIR expression was increased in TE-1/5-FU cells treated with overexpressed lncRNA HOTAIR. In addition, CCK-8 assay displayed that the depletion of lncRNA HOTAIR inhibited cell viability (*p* < 0.05) (Fig. 3f) and reduced IC_50_, which suggested that depletion of lncRNA HOTAIR promoted sensitivity and inhibited the chemoresistance of TE-1/5-FU cells to 5-FU (*p* < 0.05) (Fig. 3g). EdU assay and flow cytometry showed that silencing of lncRNA HOTAIR suppressed TE-1/5-FU cell proliferation and enhanced apoptosis (*p* < 0.05) (Fig. 3h, i), while restored lncRNA HOTAIR exerted the opposite effects. Next, we adopted PI staining using flow cytometry to verify the cell cycle of EC cells, which identified more cells at the G0/G1 phase but fewer cells in the S phase upon silencing lncRNA HOTAIR, whereas an opposite trend was observed in response to lncRNA HOTAIR overexpression (*p* < 0.05) (Fig. 3j). Thus, the findings implied that downregulation of HOTAIR promoted chemosensitivity to 5-FU and apoptosis and suppressed the proliferation of EC cells.

### HOTAIR promoted DNA methylation of MTHFR promoter

**Fig. 4:**
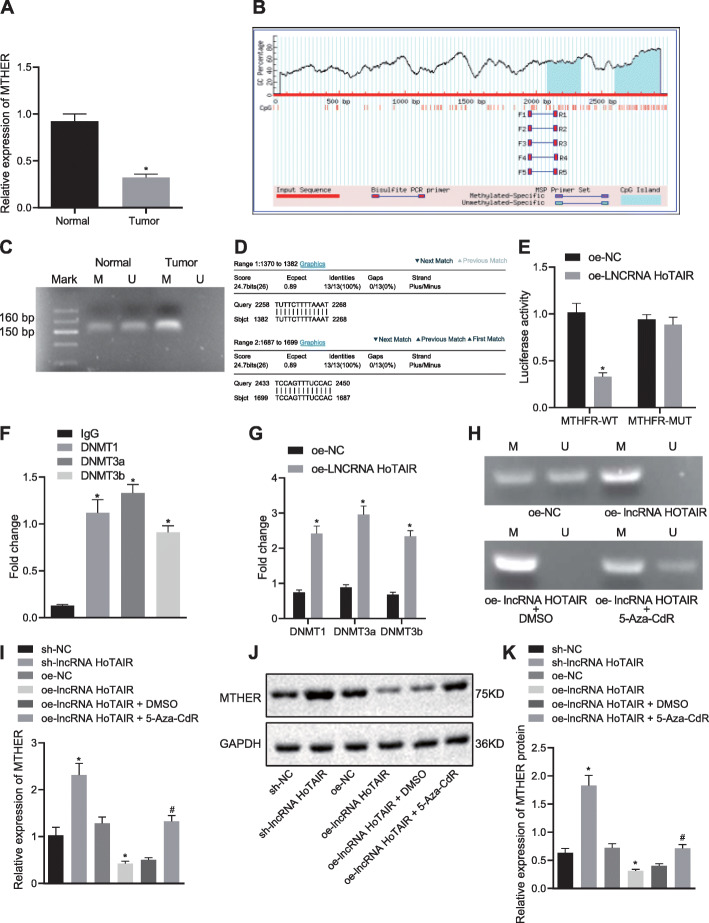
HOTAIR modulates DNA methylation within MTHFR promoter region. shRNA was delivered into TE-1 cells to specifically blunt HOTAIR (sh-lncRNA HOTAIR group). Expression vectors containing the lncRNA HOTAIR were introduced into TE-1 cells (oe-lncRNA HOTAIR group). Scramble shRNA-treated cells and empty vectors-treated cells were served as negative controls (sh-NC group and oe-NC group). Moreover, TE-1 cells treated with restored HOTAIR were added with either 5-Aza-CdR or DMSO. (**a**) MTHFR mRNA level in EC tissues (*n* = 70) and adjacent normal tissues (n = 70) determined by RT-qPCR, normalized to GAPDH. (**b**) Distribution of CpG islands within MTHFR promoter region. (**c**) MSP was adopted to detect methylation level of MTHFR promoter region in EC tissues (n = 70) and adjacent normal tissues (n = 70). (**d**) The binding sites between the lncRNA HOTAIR and MTHFR promoter regions predicted using Blast comparison website. (**e**) Target relationship verified by dual luciferase reporter gene assay. (**f**) RIP was utilized to detect the enrichment of DNA methyltransferases (DNMT1, DNMT3a and DNMT3b) affected by HOTAIR in TE-1 cells. (**g**) CHIP was employed to detect the enrichment of DNA methyltransferases (DNMT1, DNMT3a, and DNMT3b) within MTHFR promoter region in TE-1 cells. (**h**), MSP was used to detect methylation level of TE-1 cells. (**i**) MTHFR mRNA level in TE-1 cells after restoration or depletion of HOTAIR determined by RT-qPCR, normalized to GAPDH. MTHFR (75kD), GAPDH (36kD) (**j**) MTHFR protein level in TE-1 cells after restoration or depletion of HOTAIR determined by Western blot analysis, normalized to GAPDH. (**k**) Protein band patterns of MTHFR in TE-1 cells after restoration or depletion of HOTAIR determined by Western blot analysis, normalized to GAPDH. Values obtained from three independent experiments in triplicate are expressed as mean ± SD and analyzed by paired t test between EC tissues and adjacent normal tissues, by unpaired t test between EC cells and HEEC cells, and by ANOVA followed by Tukey’s post hoc test among three or more groups. *p < 0.05 compared with adjacent normal tissues, or TE-1 cells treated with IgG, oe-lncRNA HOTAIR + DMSO, sh-NC, or oe-NC group

In order to further study downstream regulatory mechanisms of lncRNA HOTAIR, RT-qPCR was adopted, which displayed that the MTHFR mRNA level was lower in EC tissues than that in adjacent normal tissues (*p* < 0.05) (Fig. 4a). MethPrimer software was utilized to predict the 4200 bp nucleotide sequence of the MTHFR promoter region and to analyze the CpG islands in its promoter region. The results confirmed that there were CpG islands in the promoter region of the MTHFR gene (Fig. 4b), indicating that MTHFR expression was reduced owing to methylation of its promoter region. In order to prove whether methylation occurred in the promoter region of the MTHFR gene in EC tissues, we detected the methylation level of CpG islands in the promoter region of MTHFR gene in 70 EC tissues and adjacent normal tissues using MSP. The results demonstrated that the methylation level of MTHFR promoter region was higher in EC tissues [methylation rate was 68.6% (48/70)] than that in adjacent normal tissues [methylation rate was 21.4% (15/70)] (*p* < 0.05) (Fig. 4c). These results suggest that the high methylation level of MTHFR led to reduction of MTHFR expression in EC.

Subsequently, we speculated whether methylation of the MTHFR promoter region was regulated by HOTAIR. In order to analyze the correlation between methylation level of MTHFR promoter region and lncRNA HOTAIR, a Blast comparison was applied to identify possible binding sites between the lncRNA HOTAIR and MTHFR promoter regions. The results showed that there were base complementary binding sites between the lncRNA HOTAIR and MTHFR promoter regions (Fig. 4d). Dual luciferase reporter gene assay displayed that the luciferase activity of MTHFR-WT was reduced in the presence of HOTAIR as compared to that of MTHFR-MUT (*p* < 0.05) (Fig. 4e), suggesting that HOTAIR can bind to the promoter region of the MTHFR gene, which was consistent with bioinformatic prediction. Further, RIP was employed to determine the enrichment of HOTAIR in relation to DNA methyltransferases DNMT1, DNMT3a and DNMT3b. The TE-1/5-FU cells overexpressing HOTAIR showed marked enrichment of HOTAIR onto the methyltransferases (Fig. 4f). In addition, CHIP was performed to determine enrichment of methyltransferases within the MTHFR promoter region, which showed that the methyltransferases were remarkably enriched in the MTHFR promoter region in TE-1/5-FU cells overexpressing HOTAIR (Fig. 4g).

In addition, MSP was utilized to assess the methylation level in the CpG site of the MTHFR promoter region in TE-1 cells with restored or depleted HOTAIR. The results indicated that the CpG sites of the MTHFR promoter region had high methylation in TE-1 cells with elevated HOTAIR (Fig. 4h), and MTHFR expression decreased (Fig. 4i-k), whereas the level of methylation was decreased following the downregulation of HOTAIR, (Fig. 4h) along with elevated levels of MTHFR (Fig. 4i-k). To further study the regulating mechanisms of HOTAIR and MTHFR, demethylation of the MTHFR promoter region was conducted using DNA methyltransferase inhibitor 5-Aza-CdR. MSP results illustrated that in TE-1 cells treated with overexpressed HOTAIR and DMSO, the MTHFR methylation level was elevated (Fig. 4h), and MTHFR expression decreased (Fig. 4i-k). Upon 5-Aza-CdR addition, the enrichment of the methyltransferases was reduced within the MTHFR promoter region in TE-1 cells (Fig. 4h), and MTHFR expression increased (Fig. 4i-k). The results revealed that the methylation level of the CpG site within the MTHFR promoter region was closely correlated with the expression of HOTAIR. In sum, the effect of HOTAIR on the methylation level of the MTHFR promoter region was exerted through DNA methyltransferases.

### MTHFR facilitated chemosensitivity of EC cells to 5-FU

**Fig. 5:**
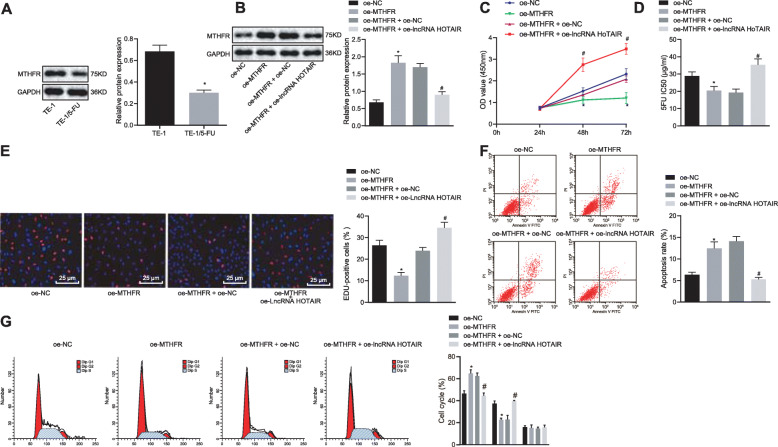
Restoration of MTHFR facilitates chemosensitivity to 5-FU and apoptosis and reduces proliferation of EC cells. Expression vectors containing the MTHFR or containing MTHFR and lncRNA HOTAIR were introduced into TE-1/5-FU cells (oe-MTHFR and oe-MTHFR + oe-lncRNA HOTAIR group). Empty vectors-treated cells were served as negative controls (oe-NC group and oe-MTHFR + oe-NC group). **a** Western blot analysis was performed to determine the MTHFR expression in TE-1 cells and TE-1/5-FU cells, normalized to GAPDH. **b** Overexpression efficiency in TE-1/5-FU cells detected using Western blot analysis. **c** CCK-8 assay was performed to detect TE-1/5-FU cell viability following MTHFR alternations. **d** CCK-8 assay was performed to measure IC_50_ value in TE-1/5-FU cells following MTHFR alternations. **e** EdU assay was performed to measure cell proliferation of TE-1/5-FU cells following MTHFR alternations (× 400). **f** Flow cytometry was performed to detect apoptosis of TE-1/5-FU cells following MTHFR alternations. **g** Flow cytometry was performed to detect cell cycle of TE-1/5-FU cells following MTHFR alternations. Values obtained from three independent experiments in triplicate are expressed as mean ± SD and analyzed by ANOVA followed by Tukey’s post hoc test among three or more groups. *p < 0.05 compared with TE-1/5-FU cells treated with either sh-NC or oe-NC group

It has been shown that MTHFR was poorly expressed in EC. In order to study the effect of MTHFR on the chemosensitivity of TE-1/5-FU cells to 5-FU, Western blot analysis was performed to measure the MTHFR protein levels in TE-1 cells and TE-1/5-FU cells, which suggested that HOTAIR exhibited a higher expression in TE-1/5-FU cells than in TE-1 cells (*p* < 0.05) (Fig. 5a). Next, TE-1/5-FU cells were treated with elevated MTHFR and/or lncRNA HOTAIR to verify the roles of MTHFR in chemosensitivity of EC cells to 5-FU. Western blot analysis revealed that MTHFR expression increased in TE-1/5-FU cells treated with overexpressed MTHFR, and decreased in TE-1/5-FU cells treated with overexpressed MTHFR and lncRNA HOTAIR (Fig. 5b). In addition, the CCK-8 assay displayed that upregulated MTHFR inhibited cell viability (*p* < 0.05) (Fig. 5c) and reduced IC_50_, and the effects of elevated HOTAIR could be reversed by overexpressed MTHFR, which suggested that upregulation of MTHFR promoted the chemosensitivity and inhibited the chemoresistance of TE-1/5-FU cells to 5-FU (*p* < 0.05) (Fig. 5d). EdU assay and flow cytometry showed that elevated MTHFR suppressed TE-1/5-FU cell proliferation and enhanced apoptosis (*p* < 0.05) (Fig. 5e, f), while the effects of elevated HOTAIR could be reversed by overexpressed MTHFR. Moreover, PI staining and flow cytometry results revealed that overexpression of MTHFR increased the cells at the G0/G1 phase, but reduced the cells in the S phase, indicating that the effects of elevated HOTAIR on EC cell cycle could be reversed by overexpressed MTHFR (*p* < 0.05) (Fig. 5g). These results confirmed that restoration of MTHFR facilitated chemosensitivity to 5-FU and apoptosis while suppressing the proliferation of EC cells.

### Silencing of HOTAIR promoted chemosensitivity of EC cells to 5-FU in vivo

**Fig. 6:**
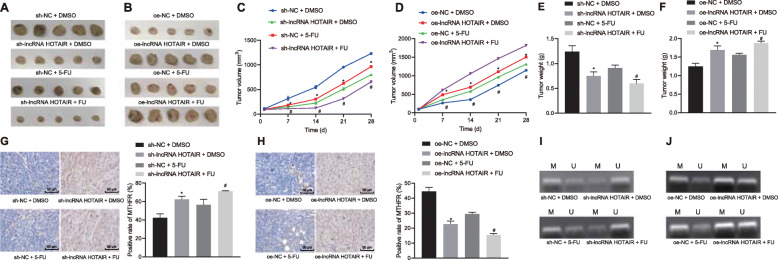
Silencing of HOTAIR elevates chemosensitivity of EC cells to 5-FU and suppresses tumor growth by elevating MTHFR in vivo*.* Nude mice were injected with TE-1 cells treated with oe-NC, oe-lncRNA HOTAIR sh-NC and sh-lncRNA HOTAIR vectors, followed by intraperitoneal injection of 5-FU and DMSO (*n* = 6). **a**, **b** Transplanted tumors of nude mice following treatment. **c**, **d** Tumor volume of nude mice following treatment. **e**, **f** Tumor weight of nude mice following treatment. **g**, **h** The positive rate of MTHFR expression in transplanted tumors following treatment, measured by Immunohistochemistry (× 200). **i**, **j** MTHFR methylation in transplanted tumor tissues following treatment, measured by MSP. Values obtained from three independent experiments in triplicate are expressed as mean ± SD and analyzed by ANOVA followed by Tukey’s post hoc test among three or more groups. *p < 0.05 compared with TE-1/5-FU cells treated with sh-NC group

To further study the regulatory effects of lncRNA HOTAIR on chemosensitivity of EC cells in vivo, TE-1 cells treated with vectors containing sh-NC and sh-lncRNA HOTAIR, oe-NC, or oe-lncRNA HOTAIR were inoculated into nude mice. Twenty-eight days after TE-1 implantation, we examined whether silencing of lncRNA HOTAIR increased the sensitivity of EC cells to 5-FU. When the size of transplanted tumors was larger than 600 mm^3^, nude mice were intraperitoneally injected with 5-FU and DMSO (as control). The results implied that tumor volume and weight significantly reduced in nude mice injected with cells treated with sh-lncRNA HOTAIR and DMSO, and 5-FU treatment further inhibited tumor growth (*p* < 0.05). However, increased tumor volume and weight were observed in nude mice injected with cells treated with oe-lncRNA HOTAIR and DMSO, whereas no marked difference was identified upon 5-FU treatment (Fig. 6a-f). Immunohistochemistry showed that the positive rate of MTHFR in tumors was significantly increased by silencing of lncRNA HOTAIR (*p* < 0.05), and an opposite trend was observed upon overexpression of lncRNA HOTAIR (Fig. 6g-h). MSP results revealed that silencing of lncRNA HOTAIR inhibited MTHFR methylation in tumor tissues, and an opposite trend was observed upon overexpression of lncRNA HOTAIR (Fig. 6j-k). These findings illustrated that HOTAIR knockdown could upregulate MTHFR to promote chemosensitivity of EC cells to 5-FU in vivo*.*

## Discussion

EC, affecting more than 450,000 people, remains the sixth leading cause of cancer-related death worldwide [4]. Despite progressive improvements in the arena of chemotherapy of EC, resistance to chemotherapy remains a stumbling block [5]. In recent years, an lncRNA, HOTAIR, has been reported to exert an anticarcinogenic effect on EC [10]. The aim of the present study was to determine the modulatory effects of silencing of HOTAIR on the cellular chemosensitivity of EC in relation to 5-FU mediated via the regulation of MTHFR methylation.

Initially, the data obtained in the present study demonstrated that HOTAIR was upregulated in EC tissues, particularly in EC tissues resistant to 5-FU. Accumulating data have similarly indicated that many lncRNAs are aberrantly expressed in various human cancers, including EC [21]. A number of lncRNAs, including HOTAIR, are reported as highly expressed in EC tissues and cells [7]. The results of the current study displayed that silencing of HOTAIR promoted chemosensitivity to 5-FU and apoptosis while repressing cell proliferation and tumor growth in EC. In general, lncRNAs have been shown to be key mediators in chemoresistance, and lncRNA LINC00261 has been reported to significantly modulate the chemoresistance observed in 5-FU in human EC [22]. Lu et al. have similarly shown that 5-FU could repress proliferation and induce the apoptosis of EC cells [5]. Interestingly, the depletion of HOTAIR has been noted to inhibit 5-FU resistance in colorectal cancer, suppress cell viability and induce G1-phase arrest of colorectal cancer cells [23]. Overexpression of HOTAIR is associated with the elevation of cell proliferation, migration, and invasion in EC, facilitating its progression and development [9]. Other co-workers have also confirmed that depletion of lncRNA HOTAIR contributes to the inhibition of cell proliferation and tumor metastasis and facilitation of apoptosis in ESCC [8, 24], corresponding with the findings of the present study. Taken together, there exists significant evidence indicating that HOTAIR is able to promote the chemosensitivity of EC cells to 5-FU by inhibiting proliferation and increasing the apoptosis of EC cells.

The present study also demonstrated that MTHFR was poorly expressed in EC tissues and cells, and elevated MTHFR enhanced chemosensitivity of EC cells to 5-FU with exhibition of lower methylation levels at the MTHFR promoter region. It has been confirmed that MTHFR is of great significance in the progression and development of EC [16]. At present, 5-FU is regarded to be the most common chemotherapeutic treatment option in colorectal cancer, and systemic exposure to 5-FU is reported as generally regulated by MTHFR [25]. A recent study has demonstrated that MTHFR could induce the sensitivity of colorectal cancer cells to 5-FU [17]. Our results imply that silencing of HOTAIR elevated MTHFR expression by decreasing MTHFR methylation. DNA methylation has been said to exert its influence on the chemosensitivity of cancers by regulating expression of genes associated with cell cycle and apoptosis [26]. The MTHFR is reported to play anti-tumor roles in human cancers through hypomethylation of DNA [27]. HOTAIR is also proved to regulate DNA methylation [14], although the targeting relationship between HOTAIR and MTHFR has not been documented earlier. Based on our findings and existing evidence, we suggest that HOTAIR expression is negatively correlated with MTHFR expression via DNA methylation. Overall, the current study has demonstrated that downregulation of HOTAIR increased MTHFR expression by decreasing MTHFR methylation, leading to an elevation in the chemosensitivity of EC cells to 5-FU.

## Conclusion

**Fig. 7:**
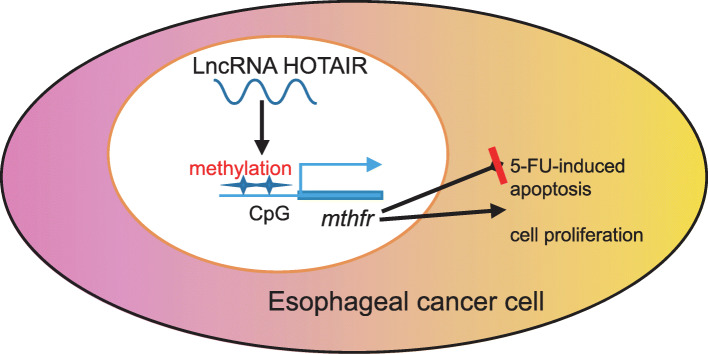
A schematic representation of the function of lncRNA-HOTAIR in the progression of EC by regulating MTHFR. LncRNA HOTAIR downregulated MTHFR expression via elevating MTHFR methylation, thus contributing to enhanced cell proliferation, but inhibited cell apoptosis and the chemosensitivity of EC cells to 5-FU

In conclusion, silencing of lncRNA HOTAIR promotes the chemosensitivity of EC cells to 5-FU and leads to abnormal cell proliferation and apoptosis of EC cells via the regulation of MTHFR methylation (Fig. 7). This identification of HOTAIR knockdown via MTHFR in mediating the chemosensitivity of EC cells to 5-FU may advance an understanding of the molecular mechanisms underpinning EC, with the potential of serving as a prognostic marker during chemotherapeutic treatment of EC. Moreover, our results provided a novel glimpse of the mechanistic link between essential non-coding RNA HOTAIR and MTHFR, and proposed the negative regulatory axis between HOTAIR/MTHFR as a promising molecular target for EC intervention. Further studies are required, however, to fully understand the specific mechanisms of HOTAIR combined with MTHFR methylation on chemosensitivity to 5-FU treatment in EC.

## Data Availability

The datasets generated and/or analysed during the current study are available from the corresponding author upon reasonable request.

## Abbreviations

EC: Esophageal cancer
5-FU: 5-fluorouracil
lncRNAs: long non-coding RNAs
DNMTs: DNA methyltransferases
MTHFR: Methylenetetrahydrofolate reductase
HOTAIR: HOX transcript antisense RNA
